# Comparative Analysis of Morphological and Release Profiles in Ocular Implants of Acetazolamide Prepared by Electrospinning

**DOI:** 10.3390/pharmaceutics13020260

**Published:** 2021-02-15

**Authors:** Mariana Morais, Patrícia Coimbra, Maria Eugénia Pina

**Affiliations:** 1 Faculty of Pharmacy of University Coimbra, Pólo das Ciências da Saúde, Azinhaga de Santa Comba, 3000-548 Coimbra, Portugal; maryrmorais@gmail.com; 2 Department of Chemical Engineering, University Coimbra, CIEPQPF, Rua Sílvio Lima, Pólo II—Pinhal de Marrocos, 3030-790 Coimbra, Portugal; patricia@eq.uc.pt; 3 FFUC, Pólo das Ciências da Saúde, Azinhaga de Santa Comba, University Coimbra, CIEPQPF, 3000-548 Coimbra, Portugal

**Keywords:** ocular implants, electrospinning technique, glaucoma, sustained drug release, poly ε-caprolactone, electrospun fibers

## Abstract

The visual impairment that often leads to blindness causes a higher morbidity rate. The goal of this work is to create a novel biodegradable polymeric implant obtained from coaxial fibers containing the dispersed drug—acetazolamide—in order to achieve sustained drug release and increase patient compliance, which is of the highest importance. Firstly, during this work, uncoated implants were produced by electrospinning, and rolled in the shape of small cylinders that were composed of uniaxial and coaxial fibers with immobilized drug inside. The fibers were composed by PCL (poly ε-caprolactone) and Lutrol F127 (poly (oxyethylene-b-oxypropylene-b-oxyethylene)). The prepared implants exhibited a fast rate of drug release, which led to the preparation of new implants incorporating the same formulation but with an additional coating film prepared by solvent casting and comprising PCL and Lutrol F127 or PCL and Luwax EVA 3 ((poly (ethylene-co-vinyl acetate)). Implants were characterized and in vitro release profiles of acetazolamide were obtained in phosphate buffered saline (PBS) at 37 °C. The release profile of the acetazolamide from coated implant containing Luwax EVA 3 is considerably slower than what was observed in case of coated implants containing Lutrol F127, allowing a sustained release and an innovation relatively to other ocular drug delivery systems.

## 1. Introduction

Visual impairment that often leads to blindness is among the diseases that causes a higher morbidity rate. According to World Health Organization (WHO) data, in 2014, 285 million people suffer from visual impairment, of which 39 million are blind and about 90% live in developing countries [1].

The conditions related to those diseases are often silent, and therefore, it is estimated that 82% of the blind are over 50 years old. Thus, "prevention" is such a fundamental role that, according to the same organization, 40% of childhood blindness is preventable or treatable.

The causes of the most common visual disabilities are refractive errors (nearsightedness, farsightedness and astigmatism), around 43% of the population. Glaucoma, macular degeneration related to age, cataracts and ocular infections are other causes of visual impairment that can lead to blindness if not properly treated [2]. The number of patients with vision-threatening conditions has been steadily increasing in recent years, largely driven by the growth and ageing of the world’s population [1].

In 2013, the WHO approved the action plan 2014–2019 for universal access to eye health, a road map for the member states, the WHO secretariat and international partners in order to achieve a measurable reduction of 25% of avoidable visual impairment in 2019.

However, the implementation and enforcement of these measures (which have difficulty getting off the paper) have not reached the best results. Improvements exist but are in amount and follow-up still limited. Given these forecasts and expectations, it has become urgent to develop systems for the diagnosis, treatment and/or maintenance of the visual system. Many of the major eye diseases of non-refractive nature are treated/controlled with drugs acting in the anterior segment of the eye, administered topically and formulated into eye drops and ointments [3,4].

Nevertheless, in the last decade, intravitreal injections of steroids have been increasingly used in the treatment of non-inflammatory diseases of the eye, which include macular edema, macular degeneration and age-related proliferative diabetic retinopathy. With respect to ocular injection, rapid movement to the posterior segment of the eye leads to decreased half-life time of the drug and concentration on site. Periodic injections are required, which not only lead to patient discomfort but also cause other complications [5], such as vitreous hemorrhage, infections, cataracts and detachment of the retina. These diseases, of which the frequency will increase in the future due to aging and lifestyle, are also chronic diseases on which traditional treatment proves ineffective [6]. It is necessary to develop alternatives to out-of-date formulations, such as sustained release systems for drugs that can be implanted close to the target tissue.

Micro- and nanoparticles, such as microspheres and liposomes, are not a viable option because its accumulation in the vitreous cavity is likely to cause blurring of vision and hinder the examination of fundus by an ophthalmologist [7]. For all this, recently, implants were developed capable of promoting the sustained release of drugs into the eye; these implants are divided into intraocular lenses for refractive error correction and drug delivery systems [8,9].

Electrospinning is a versatile methodology by which a variety of constructs can be obtained with several applications [10,11,12,13]. In the field of drug delivery, electrospun fibers evidence several advantages, such as relatively easy drug entrapment, the obtention of high drug loading and specific morphology during the process. An additional advantage is represented by the possibility of their post-treatment (which can include coating) with the possibility of modulate drug delivery, constituting an important innovation.

Acetazolamide, a carbonic anhydrase inhibitor, is still the most effective drug for the treatment of glaucoma for many years [14]. Recent attempts were made in order to develop an effective formulation that include incorporation of drug in dendritic nanoarchitectures [15] and nanoemulsions [16]. However, these efforts did not exempt the toxicity of its components, mainly in nanoemulsion formulations [17].

Regarding the previous considerations, the aim of this experimental study was to formulate novel acetazolamide polymeric implants that allow a higher sustained drug release than other drug delivery ocular systems, in order to decrease side effects and increase patient compliance.

## 2. Materials and Methods

### 2.1. Materials

Acetazolamide, purity 99%, was purchased from Alfa Aesar (Heysham, UK). Poly (ε-caprolactone), Lutrol F127 (poly (oxyethylene-b-oxypropylene-b-oxyethylene)) with 70% oxyethylene and molar mass of 9.8 to 14.6 g/mol and Luwax EVA 3 (poly (ethylene-co-vinyl acetate)) with 13–15% of vinyl acetate were purchased from BASF (Prior Velho, Portugal). Chloroform (CLF), dimethylformamide (DMF), methanol (MeOH), tetrahydrofuran (THF) and ethanol (EtOH), analytical reagents grade, were obtained from Fisher Scientific (Loughborough, UK). Reagents used in the preparation of phosphate buffered saline (PBS) were acquired from Sigma (Lisboa, Portugal).

### 2.2. Preparation of the Formulations for Electrospinning Fibrous and Coaxial Fibers

The preparation of the implant begins with the production of electrospun fibrous mats with coaxial or uniaxial fibers. Coaxial fibers consisted of a shell of PCL and a core of acetazolamide (ACZ) and Lutrol F127. The shell solutions were prepared by dissolving 1.2 g of PCL in 8 mL of CLF: DMF: 3:1 (*v*/*v*) mixture. Two solvent systems were used to prepare the cores mixtures: MeOH:DMF: 3:1 (*v*/*v*), which dissolves Lutrol F127 but only partially dissolves ACZ, and EtOH:DMF:H_2_O 2:1:1 (*v*/*v*/*v*), able to completely dissolve both Lutrol F127 and ACZ. The coaxial fibers resultant of these two different core formulations were designated as MD and EDW, following the initials of the solvents used (Table 1). Mats with fibers with a uniaxial structure were also prepared, by blending PCL and Lutrol F127 in a mixture of CLF:DMF 7:3 (*v*/*v*) (Table 1).

### 2.3. Preparation of Membranes Using an Electrospinning Setup

The electrospinning apparatus includes a high-voltage generator SL 10–300 W from Spellman, a system of coaxial needles, a copper square collector and two syringe pumps loaded with PCL solution, to form the fibers shell, and the core mixture, consisting of dissolved Lutrol F127 and dissolved or dispersed ACZ. The applied voltage, flow rate or flow delivered by each syringe and the distance between syringe tip and collector [18,19] applied are presented in Table 2.

### 2.4. Implant Preparation/Preparation of Coated Films Implants

The membranes obtained above were cut into rectangles with 4 cm long by 1.5 cm wide, approximately. The implants were obtained by rolling the referred rectangles into cylinders of 1.5 cm high. Small amounts of chloroform were used, for sealing the ends of the cylinders (the two bases) and the side part.

Some of the implants were coated with polymeric films, composed of blends of PCL and Lutrol 127 or PCL and Luwax EVA 3. The films were prepared by solvent casting, according to the formulations presented in Table 3. The solutions were subjected to stirring until completely dissolved. In case of solutions with Luwax EVA 3, it was necessary to resort to heating (50 °C) until achieve complete dissolution. Finally, the solutions were poured into petri dishes and placed to dry under a fume hood for 24 h to form the films. 

**Figure 1:**
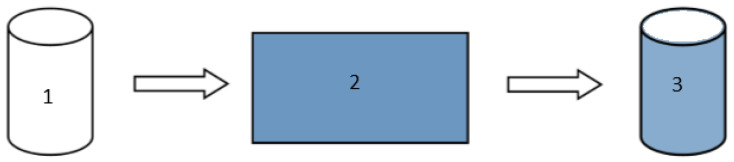
General scheme of implants coating. **1**—MD, EDW and blending implants obtained from electrospinning membranes; **2**—Polymeric films for coating composed by PCL and Lutrol F127 or Luwax EVA 3; **3**—final scheme of coated implants.

The formed films were cut into rectangles with sufficient area to cover the entire cylinder. Then, the largest edge of the rectangle was swabbed with chloroform facilitating cylinder adhesion. Finally, part of the MD and EDW implants were coated by the respective film by having their ends rolled up and sealed with heat in according with Figure 1.

### 2.5. Characterization of Implants

In the morphological and physicochemical characterization of the prepared implant, methods were used to examine the morphological characteristics of the fibers and implants, for analyzing the content of fibers and to study the hydrophilicity degree of the implants.

#### 2.5.1. Scanning Electronic Microscopy (SEM)

This method was used to access the morphology of the produced fibers, transversal surface of the coated implants and lateral surface of the same implants. Cylindrical implants were immersed in liquid nitrogen and then cut in half with a scalpel, and both sides were used in the analysis by SEM: one to observe the cross section and the other to analyze the lateral surface. The samples were glued to a holder with carbon tape, coated with gold for 10 s and analyzed on a Gemini Geiss Field Emission SEM.

#### 2.5.2. Infrared Spectroscopy (FTIR)

FTIR spectra of fibrous mats, coating films and its individual polymeric components were recorded. The solutions were poured into petri dishes and placed in the hood for 24 h. The spectra were acquired in ATR mode on a spectrometer Jasco FT/IR-4200 equipped with a Golden Gate Single Reflection Diamond ATR. The spectra were obtained at 64 scans with resolution of 4 cm^−1^, between 600 and 4000 cm^−1^.

#### 2.5.3. Contact Angle

The hydrophilicity of the fibrous mats and coating films was evaluated by water contact angle measurements. This technique measures the angle formed between the surface under study and a water droplet. Thus, it assesses the affinity degree between the surface and water and allows conclude about the hydrophilicity degree of the surface.

Measurements were made with a Dataphysics OCA-20 contact angle analyzer (DataPhysics Instruments, Filderstadt, Germany) using the sessile drop method.

#### 2.5.4. Simultaneous Thermal Analysis

This technique refers to the application of Differential Scanning Calorimetry (DSC) coupled with Thermogravimetric Analysis (TGA) and allows to identify thermal events and to determine the glass transition, melting and degradation (onset) temperatures of the polymers. Samples with a weight between 5 and 10 mg were placed in porcelain dishes and analyzed on a Q600 SDT TA Instruments, from room temperature to 500 °C, using a heating rate of 10 °C/min.

#### 2.5.5. In Vitro Drug Release Study

Each implant was placed in individual glass vials containing 4 mL of phosphate buffered saline solution (PBS) identically to others authors [20]. The flasks were kept at 37 °C in an oven with temperature control and at predetermined intervals the release medium was completely removed and replaced by the same amount of fresh PBS. These collected samples were stored in a refrigerator (± 2 °C) until quantification.

Samples were quantified by UV/VIS spectrophotometric method at a wavelength of 266 nm [20]. The calibration curve was obtained by preparing several solutions containing different drug concentrations that ranged from 5.3 ug/mL to 26.5 ug /mL in PBS.

## 3. Results

### 3.1. Obtained Implants

The implants prepared in accordance with previous description (Section 2.4) are described in Table 4. The considered parameters were: type of fibers, fiber core, fiber inner, coating and total polymer mass.

### 3.2. Characterization of Implants

#### 3.2.1. Composition/Thickness of the Coating

Table 5 shows the values of thickness of each coating film. The polymers were dissolved in the same volume of solvent (THF) and the solutions were placed in identical glass plates. Thus, solutions with more polymer mass present thicker films, which confirms the information presented in Table 5.

#### 3.2.2. SEM

**Figure 2:**
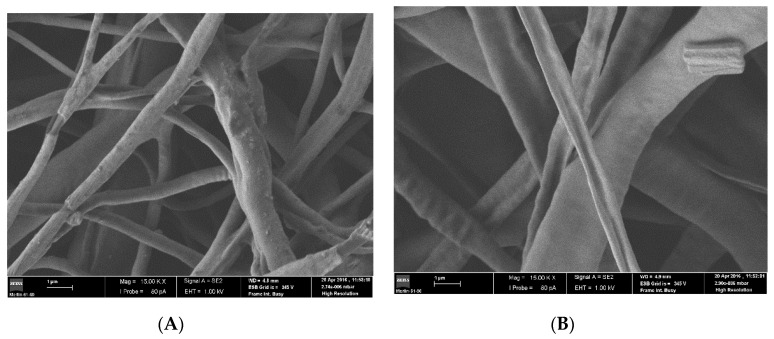
Images obtained by SEM. (**A**) Blending implant fibers, (**B**) EDW implant fibers.

Figure 2A,B represent the blending and EDW electrospun mats, respectively. It is noted that the uniaxial fibers from the blending mat have some surface beads, probably due to the accumulation of undissolved drug. The solvents of this formulation, chloroform and dimethylformamide, have a slightly hydrophobic nature, which makes it difficult to achieve complete dissolution of the drug. The possible presence of undissolved drug accumulations can cause erratic and inconstant drug release. On the other hand, the coaxial fibers from EDW implant have smooth surfaces without irregularities.

**Figure 3:**
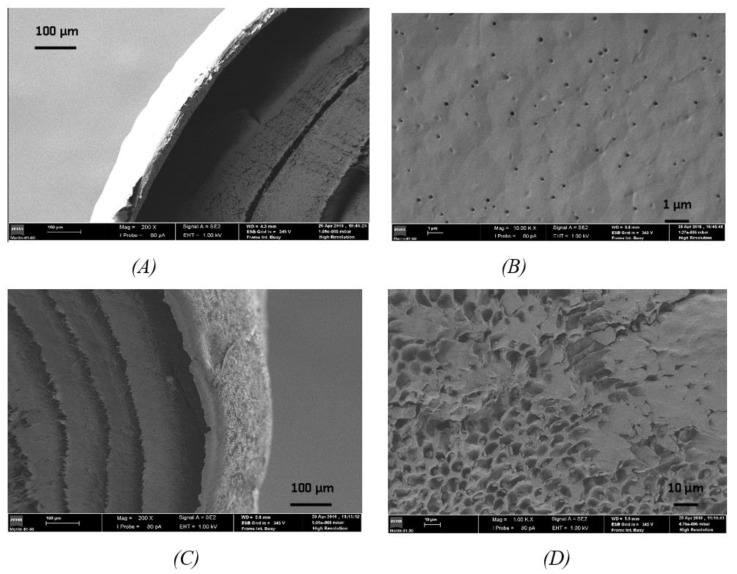
Images obtained by SEM. Cross-section view (**A**) and coating surface (**B**) of the MD 500 Lut implant and cross-section view (**C**) and coating surface (**D**) of the MD 500 Luw implant.

Figure 3 shows a cross-section and the coating surface of the MD 500 Lut and the 500 Luw MD implant, respectively. If we look at Figure 3B,D it is observed that the coating surface containing Lutrol F127 presents less porosity relative to the coating containing Luwax EVA 3, in spite of different magnification. The film containing Luwax EVA 3 had to be heated due to its low solubility in THF to achieve complete dissolution. In the cooling process, phase separation occurred: one rich in polymer and another rich in solvent. The extraction solvent by slow evaporation gave a porous structure, as can be seen in Figure 3D.

Regarding the cross-sectional view of the implant, Figure 3A,C have the same morphology: several concentric layers inside, corresponding to the wrapped fibrous membrane, and an outer layer corresponding to the coating. As for the thickness of the coating, there are no significant differences, which coincides with the results obtained through direct measurement of the thickness of the coatings shown in Table 5.

**Figure 4:**
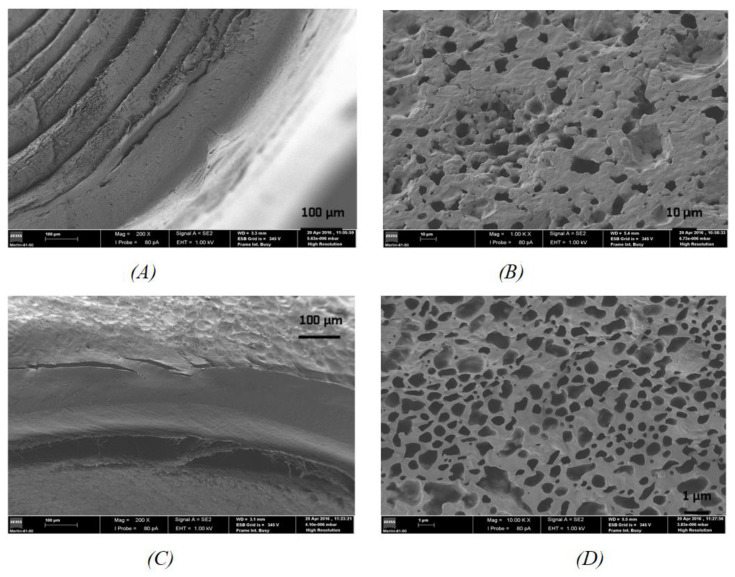
Images obtained by SEM. Cross section view (**A**) and coating surface (**B**) of the EDW 100 Lut implant and cross-section view (**C**) and coating surface (**D**) of the EDW 1000 Luw implant.

Figure 4 shows the cross-section and the coating surface of the EDW 1000 Lut and EDW 1000 Luw implants, respectively. Figure 4C,D suggest that the implant coating surface have a more extensive pore network and tubules than in the MD500 Lut and MD 500 Luw implants. In the case of the EDW 1000 Luw implant during the coating film formation, a more extensive separation occurred because of the increase in the mass of the polymer, resulting in a more irregular porous structure.

Regarding the cross-sectional view of the implants, the results are similar to earlier: several concentric layers within as a result of rolled membrane and a thicker outer coating in the case of the EDW 1000 Lut and EDW 1000 Luw implants.

#### 3.2.3. FTIR

**Figure 5:**
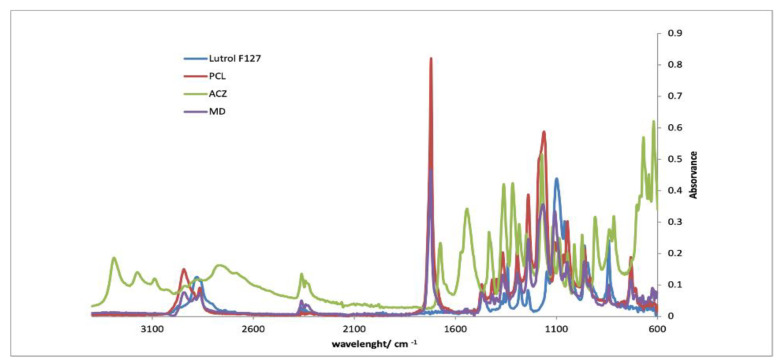
FTIR spectra of MD implants compared with PCL, Lutrol F127 and acetazolamide.

**Figure 6:**
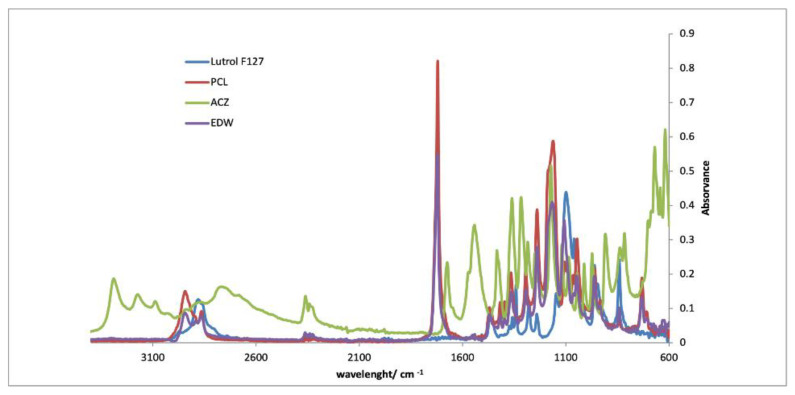
FTIR spectra of EDW implants compared with PCL, Lutrol F127 and acetazolamide.

**Figure 7:**
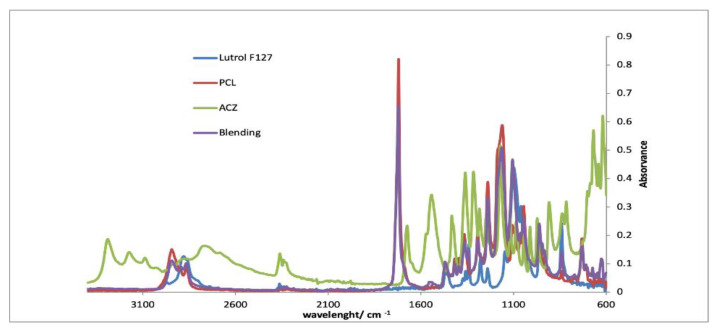
FTIR spectra of blending implants compared with PCL, Lutrol F127 and acetazolamide.

Figure 5, Figure 6 and Figure 7 show the FTIR spectra of each implant compared with the spectra of each separate component. By analyzing the spectra, it is verified that the characteristic peaks of each isolated component are visible in the spectra of the implants. It is also noticeable that the most prominent peak in the implant spectra correspond to PCL, since this is the component present in greater quantities. The results evidenced the absence of drug–polymer interactions.

#### 3.2.4. Contact Angles of Implants Surface

Analyzing Table 6 and Table 7, it appears that the contact angles vary according to the physical-chemical characteristics of materials: hydrophilic surfaces in contact with water cause the spreading of the droplet, resulting in low contact angles; on the other hand, hydrophobic surfaces in contact with water decrease the area of the contact surface, resulting in higher angles.

In Table 6, by comparing the two blending membranes, the hydrophilic character of acetazolamide is observed, causing a decrease of the contact angle.

The coaxial fibers have a higher angle than the uniaxial fibers with the drug due to the presence of PCL coating, which isolates the hydrophilic insides of the fibers.

In Table 7, it can be seen that the two coatings have different contact angles, explained by the hydrophilicity of Lutrol F127 and hydrophobicity of Luwax of EVA 3, although they represent only 25% of the coating.

#### 3.2.5. Simultaneous Thermal Analysis of the Implants

Table 8 describes the obtained parameters by analyzing the thermograms related to pure substances: acetazolamide, Lutrol F127 and PCL and membranes (EDW, MD and blending), respectively. It is noted that acetazolamide has a state transition (melting) at 274.84 °C, which is not found in the thermograms of membranes. Thus, it can be inferred that in EDW, MD and blending implants, the drug interacts strongly with Lutrol F127, which is in amorphous form or the drug amount (about 5%) is so low that the peak regarding the transition is unnoticeable.

The melting temperatures of membranes have intermediate values between the melting temperature of Lutrol F127 (60.43 °C) and PCL (67.47 °C). The same is applied to the degradation temperatures. These results suggest that the thermal events of membranes are close to the thermal events of its two major components (Lutrol F127 and PCL).

#### 3.2.6. Drug Release Assay of Implants with and without Coating

**Figure 8:**
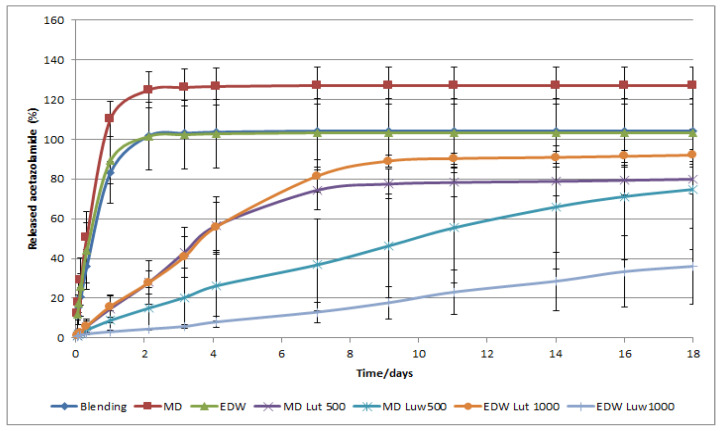
Percentage of drug release through time regarding implants with and without coating.

Through the analysis of Figure 8, it is observed that MD, EDW and blending implants without coating exhibit very similar release rates, despite previous studies showing a clear difference in release patterns between blending and coaxial fibers [18,21]. Therefore, it is concluded that, under these conditions, the type of fiber (blending or coaxial) does not affect the release of the drug. The implants without coating had the fastest release rates, i.e., during the first 48 h, they released almost all pharmacological content of the systems. It is worth noting the fact that MD implants exhibit a percentage of cumulative drug release of more than 100%, which can be explained by the acetazolamide not being totally encapsulated and instantaneously “released” and/or by the saturation of the PBS medium, with no observation of sink conditions [20].

With regard to coated implants, it is apparent that the coating containing Lutrol F127 had a faster release rate than coated implants containing Luwax EVA 3. This is explained because Lutrol F127, one hydrophilic polymer, shows higher affinity with saline medium and the diffusion of the drug through swollen polymer layer is faster; in the case of Luwax EVA 3, there is less affinity with saline medium, and its penetration in the pores with subsequent erosion and drug diffusion occurs more slowly [22].

The coating thickness strongly affects the release kinetics in case of Luwax EVA 3, as discernible in Figure 8, since the distance traveled by the drug until it reaches the implant—the diffusional distance—is greater, and therefore, the diffusion is delayed. On the other hand, the thickness of coating containing Lutrol F127 does not influence the release rate, which is explained by its swelling.

The coated implants have a more favorable release rate of acetazolamide and, indeed, suggest a promisor alternative to nanoparticles used in previous studies [23,24], whose content was released in less than 24 h.

## 4. Discussion

Summing up, seven polymeric types of acetazolamide ocular implants (with and without coating) were prepared by electrospinning/solvent casting method, respectively, in accordance with the previous description.

The characterization of prepared implants includes several studies: SEM analyses revealed some differences in structure related to the composition; however, the thickness of the coating coincides with the results obtained through direct measurements. FTIR results indicate compatibility drug-polymers. Contact angles of implants surface change according to the nature of surface (hydrophilic or hydrophobic) in contact with water. Thermal analysis of the implants suggested that the observed thermal events are related to the thermal events of its two major polymers (Lutrol 127 and PCL). These results are in line with the results obtained by other researchers [20].

In order to mimic the pH value of 7.4 of the human eye, the devices were placed in falcons with PBS, and for the test to take place at body temperature and consequently the human eye, the falcons were placed in an oven at 37 °C; at pre-determined intervals, the entire volume of PBS was removed from the falcons, and replaced with a new solution of PBS, to mimic the in vivo simulated condition for the drug release study from ocular implants. All implants have been subjected to release tests for 18 days. Through the release profile of each type of implant and their morphological and physicochemical features, it was concluded that the implants exhibiting an effective performance in drug release are constituted of PCL and Lutrol F127 dissolved in ethanol, dimethylformamide and water-EDW formulation—and coated with Luwax EVA 3. The presence of water as a solvent of Lutrol F127 and acetazolamide caused a more effective dissolution leading to a more uniform and gradual release rate. Moreover, the hydrophobicity of Luwax EVA 3 is related to polymer degradation by erosion, which retards the drug release. On the other hand, the devices with thicker coating, namely, the obtained film by dissolving 1000 mg of Luwax EVA 3 in 10 mL of THF, led to a slower release, since the distance that separates the drug from the saline medium is greater, motivating a delay in its diffusion.

The coated implants have a more favorable release rate of acetazolamide than nanoparticles used in previous studies, in which the content was released in less than 24 h [23,24].

Previous studies have demonstrated that these drug delivery systems can be used in vivo; examples are the approved FDA Iluvien^®^ and Ozurdex^®^ systems. The core-shell fibers appear as an improvement of this system combining the erosion (phenomenon that mainly happens in the Ozurdex^®^) with the diffusion (phenomenon that mainly happens in Iluvien^®^) [5]. By controlling the thickness of the implant cover, we may in the future modulate the drug release in according with the required objectives.

We cannot fail to mention that sterilization of ocular implants is inevitable to avoid infection after surgery. However, sterilization methods have to be reliable and chosen according to the used materials properties. In one recent study carried out by Cassan et al. [25], the impact of three different sterilization techniques (β, γ and X-ray) on electrospun PCL scaffolds was evaluated. It was shown that material properties of PCL in electrospun fiber mats were affected by all three sterilization techniques, although electron beam had less of an effect on PCL than y-irradiation, being recommended for PCL fiber mats.

Another experimental work [26] compared different sterilization/disinfection methods (gamma irradiation, UV-irradiation, in situ generated chlorine gas, low-temperature argon plasma) on various properties (drug stability, solid state, mechanical properties, swelling and biodegradation, drug release) of electrospun drug-loaded matrices. Two different polymeric compositions were studied—polycaprolactone (PCL) alone or in combination with polyethylene oxide (PEO)—with the model antibacterial drug chloramphenicol (CAM). Although no changes in the morphology were seen, the most crucial was the effect of sterilization on the polymer crystallinity, which is also directly changing the mechanical properties and polymer degradation behavior. The authors concluded that no single sterilization method can be considered appropriate for all materials and formulations; thus, a case-by-case approach needs to be taken when developing a novel electrospun drug delivery system as a drug product.

Paying attention to these considerations, in the near future, we will dedicate our research work to the selection of the most appropriate sterilization method for the prepared ocular implants.

## 5. Conclusions

Electrospinning is a simple and versatile method to produce fibers using charged polymer solutions. As drug delivery systems, electrospun fibers are an excellent choice because of easy drug entrapment, high surface area, morphology control and biomimetic characteristics. Various drugs and biomolecules can be easily encapsulated inside or on the fiber surface, either during electrospinning or through post-processing of the fibers. Multicomponent fibers have attracted special attention because new properties and morphologies can be easily obtained through the combination of different polymers.

Considering that acetazolamide, by inhibiting a large-scale enzyme, carbonic anhydrase, fell into disuse, mainly because of renal side effects, we can conclude that the use of these local and specialized systems allowed the application of this drug, which is effective and safe when directly administered to the vitreous humor. The continued apprehension of these systems leads to visible improvements in the quality of life of people with glaucoma.

In future studies, acetazolamide polymeric implants prepared by electrospinning and coated by a solvent casting method could be successfully used for ocular administration for the treatment of ocular affections, increasing patient compliance and resulting in visible improvements in their quality of life.

## Figures and Tables

**Table 1:** Composition of the formulations used to produce the core of the coaxial fibers and the uniaxial fibers inner.

Type	Formulation	Polymer (Mass)	ACZ (Mass)	Solvent	Volume
Coaxial	MD	0.3 g Lutrol	75 mg	MeOH:DMF 3:1	2 mL
Coaxial	EDW	0.3 g Lutrol	75 mg	EtOH:DMF:H_2_O 2:1:1	2 mL
Uniaxial	Blending	0.3 g Lutrol +1.2 g PCL	75 mg	CLF:DMF 7:3	10 mL

**Table 2:** Electrospinning parameters for each formulation.

Formulation	Lutrol F127 (Flow Rate)	PCL (Flow Rate)	Voltage	Distance to the Collector
MD	0.5 mL/h	2.0 mL/h	16 kV	20 cm
EDW	0.5 mL/h	2.0 mL/h	15 kV	20 cm
Blending	2.5 mL/h		11 kV	20 cm

**Table 3:** Coating films composition (THF: tetrahydrofuran; Lt: Lutrol F127; Lw: Luwax EVA 3).

Form	Polymers		Total (mg)	Solvent	Lt/Lw (%)	PCL (%)
500 Lut	125 mg Lutrol F127	375 mg PCL	500	THF	25%	75%
500 Luw	125 mg Luwax EVA 3	375 mg PCL	500	THF	25%	75%
1000 Lut	250 mg Lutrol F127	750 mg PCL	1000	THF	25%	75%
1000 Luw	250 mg Luwax EVA 3	750 mg PCL	1000	THF	25%	75%

**Table 4:** Types of implants and their composition.

Name	Type of Fibers	Fiber Core	Fiber Inner	Coating	Total Polymer Mass
MD	Coaxial	PCL	Lutrol F127 and ACZ in Methanol and dimethylformamide (DMF)	No	-------
EDW	Coaxial	PCL	Lutrol F127 and ACZ in Ethanol, DMF and water	No	-------
MD Lut500	Coaxial	PCL	Lutrol F127 and ACZ in Methanol and DMF	Lutrol F127 and PCL polymer	500 mg
MD Luw500	Coaxial	PCL	Lutrol F127 and ACZ in Methanol and DMF	Luwax EVA 3 and PCL polymer	500 mg
EDW Lut1000	Coaxial	PCL	Lutrol F127 and ACZ in Ethanol, DMF and water	Lutrol F127 and PCL polymer	1000 mg
EDW Luw1000	Coaxial	PCL	Lutrol F127 and ACZ in Ethanol, DMF and water	Luwax EVA 3 and PCL polymer	1000 mg
Blending	Uniaxial	Lutrol F127 and ACZ			No

**Table 5:** Composition and thickness of coating films.

Solutions	Polymers (Weight)		Total (Weight)	Thickness (µm)
500 Lut	125 mg Lutrol F127	375 mg PCL	500 mg	52.5 ± 12.6
500 Luw	125 mg Luwax EVA 3	375 mg PCL	500 mg	50.0 ± 8.1
1000 Lut	250 mg Lutrol F127	750 mg PCL	1000 mg	168.5 ± 6.2
1000 Luw	250 mg Luwax EVA 3	750 mg PCL	1000 mg	271.3 ± 6.3

**Table 6:** Water membrane contact angles (Mean ± SD, *n* = 3).

Membranes			
Blending without ACZ	Blending	MD without ACZ	MD
Angle/	Angle/	Angle/	Angle/
58.84 ± 10.60	38.08 ± 12.16	100.35 ± 4.58	50.75 ± 4.45

**Table 7:** Water-coating contact angles (Mean ± SD, *n* = 3). Simultaneous Thermal Analysis of the Implants.

Coating	
PCL-Lut 75:25	PCL-Luw 75:25
Angle/	Angle/
37.22 ± 4.40	102.60 ± 3.40

**Table 8:** Melting temperature, onset degradation temperature and variation of melting enthalpy of pure substances: acetazolamide, Lutrol F127, PCL and the membrane formulations: EDW, MD and blending.

Substance/ Membrane	Melting Temperature (°C)	Variation of Melting Enthalpy (J/g)	Onset Degradation Temperature (°C)
Acetazolamide	274.84	−132.20	271.25
Lutrol F127	60.43	−123.50	370.30
PCL	67.47	−67.34	388.27
EDW	61.03	−54.36	379.89
MD	61.85	−57.71	380.03
Blending	61.78	−61.30	382.79

## Data Availability

Not applicable.
